# Use of a rapid recombinase-aided amplification assay for *Mycoplasma pneumoniae* detection

**DOI:** 10.1186/s12879-019-4750-4

**Published:** 2020-01-28

**Authors:** Guanhua Xue, Shaoli Li, Hanqing Zhao, Chao Yan, Yanling Feng, Jinghua Cui, Tingting Jiang, Jing Yuan

**Affiliations:** 1 Department of Bacteriology, Capital Institute of Pediatrics, No. 2 Yabao Road, Chaoyang District, Beijing, 100020 China; 2 Department of Obstetrics, Southern District of the Fifth Medical Center of PLA General Hospital, No. 8 Dongdajie Road, Fengtai District, Beijing, 100071 China

**Keywords:** *Mycoplasma pneumoniae*, Recombinase, Recombinase-aided amplification, Detection, Molecular diagnostic technique

## Abstract

**Background:**

*Mycoplasma pneumoniae* is one of the most common causative pathogens of community-acquired pneumonia (CAP), accounting for as many as 30–50% of CAP during peak years. An early and rapid diagnostic method is key for guiding clinicians in their choice of antibiotics**.**

**Methods:**

The recombinase-aided amplification (RAA) assay is a recently developed, rapid detection method that has been used for the detection of several pathogens. The assays were performed in a one-step single tube reaction at 39° Celsius within 15–30 min. In this study, we established an RAA assay for *M. pneumoniae* using clinical specimens for validation and commercial real-time PCR as the reference method.

**Results:**

The analytical sensitivity of the RAA assay was 2.23 copies per reaction, and no cross-reactions with any of the other 15 related respiratory bacterial pathogens were observed. Compared with the commercial real-time PCR assay used when testing 311 respiratory specimens, the RAA assay obtained 100% sensitivity and 100% specificity with a kappa value of 1.

**Conclusions:**

These results demonstrate that the proposed RAA assay will be of benefit as a faster, sensitive, and specific alternative tool for the detection of *M. pneumoniae*.

## Background

*Mycoplasma pneumoniae* is a major cause of community-acquired pneumonia (CAP) in adults and children, with an epidemic occurring every 3–7 years [1]. This organism can cause up to 20–40% of CAP in the general population during epidemics, and this can rise to as much as 70% in closed populations [1–3]. Its clinical manifestations range from mild bronchitis to severe pneumoniae. Asthma, chronic obstructive pulmonary disease, as well as extra-pulmonary pathologies of the joints, kidney, pancreas, liver, skin, cardiovascular system, and central nervous system, are often implicated [4]. In recent years, there have been increasing reports of severe cases of *M. pneumoniae*-related pneumonia [5]. It is, therefore, important to develop an efficient diagnostic method to guide timely clinical treatment.

Traditional detection methods for *M. pneumoniae* rely on culture, PCR or serology testing [6]. Cultures provide irrefutable evidence of infection, but as a fastidious bacteria, it requires several days to grow; thus, culturing is seldom used in clinical practice except when obtaining isolates to determine antimicrobial susceptibility or to perform other scientific research [7, 8]. Serology testing is currently the most commonly used method in many primary hospitals for its convenience, but this method produces inconsistent results when using serum samples obtained at different times during the onset of illness and when different commercial testing kits are used. For some individuals, antibodies toward *M. pneumoniae* persist for a long time, making it difficult to confirm whether a current *M. pneumoniae* infection is actually present [9, 10]. PCR is a well-developed method with several advantages over serology, and it has dramatically improved diagnostic testing due to its superior analytical and clinical sensitivity; however, PCR requires expensive instruments and skilled operators, which limit its use in small clinical laboratories [11, 12]. Now many new technical are used in *M.pneumoniae* detection, like loop-mediated isothermal amplification (LAMP), Dual-priming isothermal amplification (DAMP) and recombinase-aided amplification (RAA) assay [13–18].

The recombinase-aided amplification (RAA) assay is a new isothermal amplification technology with the advantages of rapidity, simplicity, and low cost, and it is, therefore, potentially very suitable for clinical application. In this method, the recombinase UvsX (from *E. coli*), a single-stranded DNA-binding protein (SSB), and a DNA polymerase are combined in an RAA reaction system. The UvsX recombinase and primers form a protein-DNA complex that can bind to homologous sequences in the double-stranded DNA target. Once the homologous sequence is located by the primer, a chain exchange reaction will occur to form and initiate DNA synthesis, and the target region on the template will be exponentially amplified. The amplification process is completed within 15–30 min at 39 °C [13, 14]. Several reports have confirmed the successful application of this technology to the detection of a variety of pathogens ([13–16], Table 1).

**Table 1:** The diagnostic accuracy of RAA assay for other pathogens

Pathogen	sensitivity	specificity	Reference
Hepatitis B virus (HBV)	95.7%	100%	13
Coxsackievirus A6 (CVA6)	100%	98,7%	14
Coxsackievirus A10 (CVA10)	95%	99.0%	14
Respiratory syncytial virus (RSV)	100%	100%	15
human adenovirus (HDV)	100%	100%	16

In this study, we aimed to develop an RAA assay for the detection of *M. pneumoniae*. The analytical specificity and sensitivity of the assay were evaluated. Clinical samples were tested, and the results were compared with those obtained using commercial real-time PCR assays as the reference method.

## Methods

### Clinical samples

A total of 311 respiratory clinical samples (213 were BAL, 90 were sputum and 8 were swab) were collected from patients with respiratory infections at the Capital Institute of Pediatrics from October 2018 to March 2019. Among these, 141 (46.08%) were female and 165 (53.92%) were male. Their age ranged from 15 days to 16 years old. The diagnoses were 258 cases of pneumonia, 39 of bronchopneumonia, 6 of capillary bronchitis, and 8 of respiratory tract infections.

### DNA extraction

Total DNA was extracted from 200 μL of each clinical sample with the QIAamp DNA Mini Kit (Qiagen, Hilden, Germany) according to the manufacturer’s instructions. The DNA samples were eluted in 150 μL of nuclease-free water and stored at − 80 °C until use.

### Primer and probe design

The *M. pneumoniae* Reference Strain M129 (ATCC 29342) sequence was downloaded from the GenBank database (https://www.ncbi.nlm.nih.gov/pubmed). The P1 gene was chosen as the target region and all the P1 sequences available for *M. pneumoniae* were obtained from the NCBI database (https://www.ncbi.nlm.nih.gov/pubmed). The primers and probe were designed within the conserved regions according to the principles of RAA primer and probe design (Table 2). Primer-BLAST of NCBI was used to confirm the specificity of the primers and probe. The online OligoEvaluator software (http://www.oligoevaluator.com) was used to analyze the potential for primer dimers and hairpins. The primers and probe were synthesized by Sangon Biotech (Shanghai, China).

**Table 2:** Sequences of the primers and probes used for the RAA assay

Primer/probe	Sequence (5′-3′)	Genomic position	Product size (bp)
F-primer	CTTTAACAATAACCGCTGGTTTGAATATGTA	182,540–182,570	164 bp
R-primer	CTACTAAGTTCAGGTTGCTTTCAAGTTCAT	182,703–182,674	164 bp
Probe	TTGCTGGCGCTAAGTTCGTTGGTAGGGAAC [FAM-dT] C [THF] T [BHQ-dT] TTAGCGGGTACCATTA [3′-block]	182,584–182,634	164 bp

*FAM* 6-carboxyfluorescein, *THF* Tetrahydrofuran, *BHQ* Black hole quencher, *C3-spacer* 3′-phosphate blocker

### Construction of the recombinant plasmid

Recombinant plasmids containing a 450-bp fragment of the P1 gene (nt 180,858–185,741, GenBank accession no. U00089.2) were prepared. The primers used to construct the plasmid are listed in Table 2. The resulting PCR products from the P1 gene were cloned into a T-vector using the pGM-Simple-T-Kit (TIANGEN Biotech (Beijing) Co., China) to construct the recombinant plasmids. The plasmid DNA was quantified with a NanoDrop 2000 spectrophotometer (Thermo Fisher Scientific, Dreieich, Germany). The DNA copy number was calculated using the following formula: DNA copy number (copy number/μL) = [6.02 × 10^23^ × plasmid concentration (ng/μL) × 10^− 9^]/[DNA length (nt) × 660] [14]. The constructed plasmids were verified by sequencing and stored at − 20°C until use.

### RAA assay

The RAA assays were performed in 50-μL reaction volumes using a commercial RAA kit (Qitian, Jiangsu, China). The reaction mixtures contained 2 μL of extracted DNA template, 25 μL of reaction buffer, 15.7 μL of DNase-free water, 2.1 μL of primer F (10 μM), 2.1 μL of primer R (10 μM), 0.6 μL of the probe (10 μM), and 2.5 μL of 280 mM magnesium acetate. The reaction mixture was added to a tube containing the RAA enzyme mix (SSB, 800 ng/μL; UvsX, 120 ng/μL; DNA polymerase, 30 ng/μL) in a lyophilized form. The tube lids were carefully closed and the contents were mixed well before being transferred to the detection equipment, as the efficiency of the mixing process has an impact on the results. The tubes were then transferred to a QT-F7200–0001 fluorescence detector (Jiangsu, Qitian) at 39.0 °C for 20 min. A positive control (*M. pneumoniae* recombinant plasmid) and a negative control (blank, buffer only) were included in each run.

### Real-time PCR assay

A commercial detection kit for *Mycoplasma pneumoniae* DNA (based on PCR and fluorescence detection) was purchased from Mole Bioscience (Jiangsu, China). The detection kit contained specific primers and probes (P1 gene as the target), Tris-HCl buffer, Hot Start Taq enzyme, and dNTPs. The reaction mixtures contained 5 μL of extracted DNA template, 6 μL of reaction buffer, 11.5 μL of DNase-free water, 2.0 μL primer and probe, and 0.5 μL of Hot Start Taq enzyme. The cycling profile was as follows: 50 °C for 2 min and 95 °C for 2 min, followed by 40 cycles of denaturation at 91 °C for 15 s and annealing/extension at 64 °C for 1 min.

### Analytical sensitivity, specificity, and reproducibility of the RAA assay

The analytical sensitivity of the RAA assay was determined using 10-fold serial dilutions of the recombinant plasmid ranging from 10^4^ to 10^0^ copies/μL. A total of 50 *M.pneumoniae* strains were recovered for the specificity of the RAA assay which were collected from patients with respiratory infections at the Capital Institute of Pediatrics from 2016 to 2018. The assay specificity was also evaluated by testing other mycoplasma and bacterial infection samples containing either *Mycoplasma genitalium* (ATCC 33530), *Mycoplasma hominis* (ATCC 23114), *Mycoplasma penetrans* (ATCC 55252), *Mycoplasma fermentans* (ATCC 19989), *Mycoplasma hyorhinis* (ATCC 17981), *Ureaplasma urealyticum* (ATCC 27618). *Staphylococcus aureus* (ATCC 25923), *Staphylococcus epidermidis* (ATCC 12228), *Klebsiella pneumoniae* (ATCC 27736), *Streptococcus pneumoniae* (ATCC 49619), *Escherichia coli* (ATCC 25922), *Legionella pneumophila* (ATCC 33152), *Haemophilus influenzae* (ATCC 43065), *Mycobacterium tuberculosis* (ATCC 25618/H37Rv) or *Pseudomonas aeruginosa* (ATCC27853). In addition, for the detection of serial diluted recombinant plasmid, twelve replicates were performed on five separate days to validate the reproducibility of the RAA assay.

### Evaluation of the RAA assay using clinical samples

To evaluate the performance of the RAA assays for *M. pneumoniae* detection, 311 clinical samples were tested. The performance of the RAA assay was compared with that of a commercial real-time PCR assay for *M. pneumoniae*.

### Statistical analysis

Probit analysis for the detection limit of the RAA assay was performed at a 95% probability level. The kappa and *p* values of the RAA and real-time PCR assays were calculated. All statistical analysis was carried out with SPSS 21.0 (IBM, Armonk, NY).

## Results

### Analytical specificity of the RAA assay

**Fig. 1:**
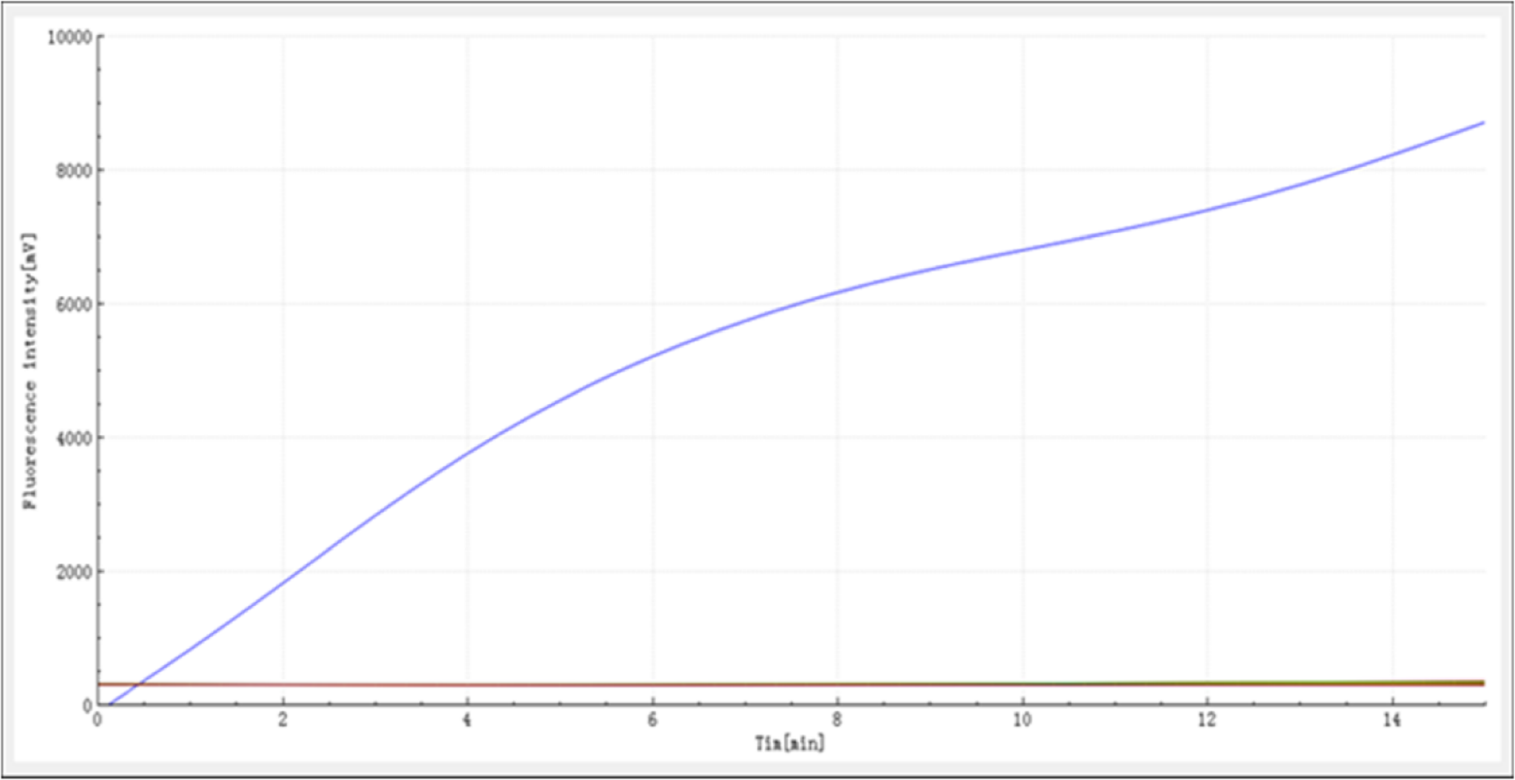
Specificity of the RAA assay for *M. pneumoniae* detection. Only the *M. pneumoniae* samples produced amplification signals, whereas the negative control (buffer only) and control bacterial samples produced negative amplification signals

The RAA assay of all known 50 *M. pneumoniae* strains showed positive, whereas all the negative control (buffer only) and the 15 control bacterial samples were negative (Fig. 1). Thus, the RAA assay for the detection of *M. pneumoniae* demonstrated high specificity (100%).

### Analytical sensitivity of RAA

**Fig. 2:**
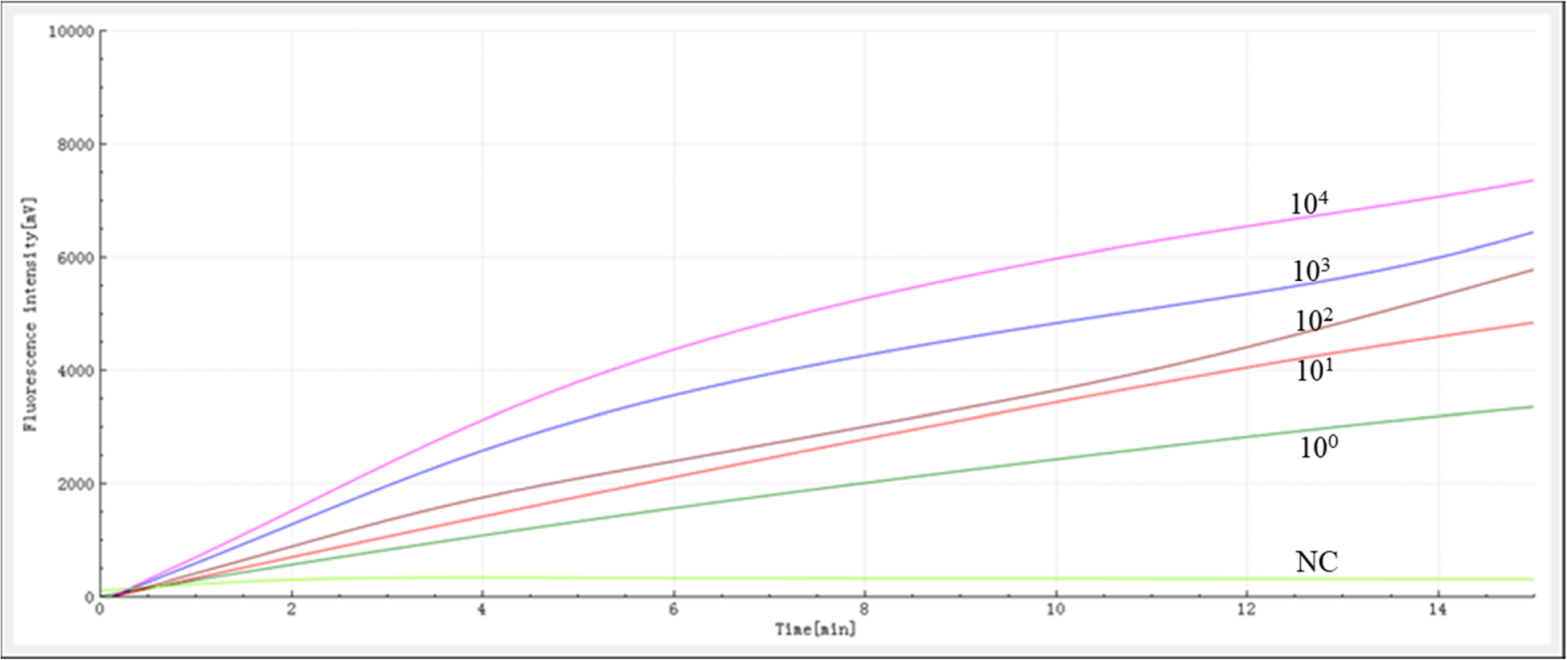
Sensitivity of the RAA assay for *M. pneumoniae* detection. A serial dilution of the recombinant plasmid was used ranging from 10^4^ to 10^0^ copies/reaction. A negative control (buffer only) was also assayed

The sensitivity of the RAA assay for *M. pneumoniae* detection was determined using a panel of serially diluted recombinant plasmids containing a fragment of the P1 gene and compared with the real-time PCR. All replicate dilutions of the recombinant plasmid from 10^4^ to 10^1^ copies per reaction produced a positive signal in the RAA assay, while 11/12 replicates containing 10^0^ copies per reaction tested positive (Fig. 2, Table 3). The detection limit of the RAA assay at 95% probability was 2.23 copies (19.4 fg) per reaction while the real-time PCR was 22.3 copies (194 fg) (probit analysis, *p* = 0.006).

**Table 3:** Reproducibility of the RAA assay and real-time PCR assay

Serial diluted DNA	No. of replicates tested/No. of detection	
	RAA	Real-time PCR
10^4^	12/12	12/12
10^3^	12/12	12/12
10^2^	12/12	12/12
10^1^	12/12	10/12
10^0^	11/12	0/12

### Evaluation of RAA assay using clinical samples and comparison with real-time PCR

A total of 311 clinical samples were used for evaluation of the RAA assay, and the results were compared with those obtained with real-time PCR as the reference method. Real-time PCR tested 101/311 samples as positive, and the RAA assay correctly identified and differentiated all 101 of these positive samples with 100% accordance, sensitivity, and specificity (Table 4). No significant differences between the detection results of RAA and real-time PCR were observed. The kappa value of the RAA assay was 1.0 (*p* < 0.001).

**Table 4:** The clinical performance of RAA for the detection of respiratory specimens of *M. pneumoniae* compared with real-time PCR as the reference method

Real-time PCR	RAA		Total	Performance of RAA compared with real-time PCR			
	Positive	Negative		Sensitivity (%)	Specificity (%)	Accordance rate (%)	Kappa value (κ)
Positive	101	0	101	100	100	100	1
Negative	0	210	210	–	–	–	–
Total	101	210	311	–	–	–	–

## Discussion

*M. pneumoniae* is an important pathogen that causes respiratory disease in adults and children. A rapid and convenient diagnostic tool is critical for clinical diagnosis and treatment and to prevent further spread of disease. Currently, the two most commonly used methods in clinical practice for *M. pneumoniae* detection are serology and real-time PCR [12]. Before PCR became widely used in clinical practice, serology was the primary means for laboratory diagnosis, even with its significant limitations such as high false-negative and false-positive rates, because there was no other rapid test available. Real-time PCR has since emerged as an ideal detection method but it remains difficult to implement in poorly funded laboratories with limited equipment and skilled personnel [19].

The RAA assay developed in the present study can be completed at a constant temperature of 39 °C in 15–30 min, which provides a significant saving in turnaround time compared with the other methods. Furthermore, the RAA detection system does not require a sophisticated laboratory setting, skilled personnel or expensive equipment, and it can be performed with a portable device. The running costs for RAA are also relatively lower than for real-time PCR. Therefore, it is a rapid and easy method for clinical use especially in primary laboratories if accompanied with a simple DNA extraction method.

The genome of *M. pneumoniae* is relatively conservative but genetic diversity among strains has been demonstrated [20]. Sequence variation within the target region can affect the detection results of molecular diagnostic methods. Previously reported targets for *M. pneumoniae* include 16S rRNA, P1, RepMP1, and the CARDS toxin [21–24]. Among these, the P1 gene has offered high specificity and diagnostic efficiency. In the present study, the RAA primers and probe were, therefore, designed based on the P1 gene, and we observed no cross-reactions with other respiratory pathogens.

In this study, the analytical sensitivity of the assay for *M. pneumoniae* was 2.23 copies (19.4 fg) per reaction, while the sensitivity of the commercial real-time PCR assay used here for comparison is only 500 copies per reaction according to the manufacturer, suggesting that the RAA assay is more sensitive. Our RAA assay also achieved the same level of sensitivity reported for real-time PCR methods, which ranges from 0.83 fg–100 fg per reaction [21–24].

To validate the clinical application of the RAA-based method, we tested 311 clinical samples. The agreement between the RAA and real-time PCR assays was 100%, suggesting that the RAA developed in the present study were suitable for diagnosing *M. pneumoniae* infections in children.

A similarly rapid detection method for *M. pneumoniae* known as loop-mediated isothermal amplification (LAMP) has been shown to have comparable sensitivity to RAA [25–27]. However, the current LAMP methods, especially LAMP with two loop primers, suffer from undesired nonspecific amplification with strong background signals due to the increasing target sites. This nonspecific amplification substantially reduced the reliability of LAMP and limited its applications in clinical diagnostics [17]. Dual-Priming Isothermal Amplification (DAMP) is another promising and simple method for rapid detection of pathogens. It was proved to have better effect to detect to detect HIV-1 DNA/RNA and *Escherichia coli* DNA, showing equal or better sensitivity with shorter detection time compared to conventional LAMP and PCR methods [18], which could be also used for *Mycoplasma pneumoniae* detection in the further study.

However, the RAA assay still has some limitations. Firstly, multiplex amplification of different targets in RAA is currently difficult because the primers for RAA each require more than 30 bp of complementary sequence and the probe requires around 50 bp, the more and longer primers in one tube will cause non-specific amplification which limits the development of a multiplex RAA assay. Secondly, RAA does not differentiate between colonization and real infection, nor a coinfection with other pathogens. This latter could be identified with the commercial multiplex PCR respiratory panel which includes *Mycolasma* species among several other bacterial and viral pathogens.

## Conclusions

The developed RAA assay exhibited high specificity and sensitivity and provides a simple, rapid, and reliable method for *M. pneumoniae* detection. The features of the developed RAA assay make this assay suitable for application toward the rapid detection of *M. pneumoniae* in underequipped diagnostic laboratories if accompanied with a simple DNA extraction method, which may prove a great help for future clinical detection and treatment in primary hospitals.

## Data Availability

The datasets used and/or analyzed during the current study available from the corresponding author on reasonable request.

## Abbreviations

CAP: Community-acquired pneumonia
LAMP: Loop-mediated isothermal amplification
RAA: Recombinase aided amplification
SSB: Single strand DNA binding protein

## References

[1] Waites KB, Xiao L, Liu Y, Balish MF, Atkinson TP (2017). Mycoplasma pneumoniae from the respiratory tract and beyond. Clin Microbiol Rev.

[2] Jacobs E, Ehrhardt I, Dumke R (2015). New insights in the outbreak pattern of mycoplasma pneumoniae. Int J Med Microbiol.

[3] Loens K, Goossens H, Ieven M (2010). Acute respiratory infection due to *Mycoplasma pneumoniae*: current status of diagnostic methods. Eur J Clin Microbiol Infect Dis.

[4] Waites KB, Talkington DF (2004). *Mycoplasma pneumoniae* and its role as a human pathogen. Clin Microbiol Rev.

[5] Ding Y, Chu C, Li Y, Li G, Lei X, Zhou W, Chen Z (2018). High expression of HMGB1 in children with refractory mycoplasma pneumoniae pneumonia. BMC Infect Dis.

[6] Waites KB, Balish MF, Atkinson TP (2008). New insights into the pathogenesis and detection of mycoplasma pneumoniae infections. Future Microbiol.

[7] Atkinson TP, Balish MF, Waites KB (2008). Epidemiology, clinical manifestations, pathogenesis and laboratory detection of mycoplasma pneumoniae infections. FEMS Microbiol Rev.

[8] CLSI (2011). Methods for antimicrobial susceptibility testing of human mycoplasmas. Approved guideline. CLSI document M43-A.

[9] Loens K (2016). Ieven M1Mycoplasma pneumoniae: current knowledge on nucleic acid amplification techniques and serological diagnostics. Front Microbiol.

[10] Busson L, Van den Wijngaert S, Dahma H, Decolvenaer M, Di Cesare L, Martin A, Vasseur L, Vandenberg O (2013). Evaluation of 10 serological assays for diagnosing mycoplasma pneumoniae infection. Diagn Microbiol Infect Dis.

[11] Kashyap B, Kumar S, Sethi GR, Das BC, Saigal SR (2008). Comparison of PCR, culture & serological tests for the diagnosis of mycoplasma pneumoniae in community-acquired lower respiratory tract infections in children. Indian J Med Res.

[12] Chang HY, Chang LY, Shao PL, Lee PI, Chen JM, Lee CY, Lu CY, Huang LM (2014). Comparison of real-time polymerase chain reaction and serological tests for the confirmation of mycoplasma pneumoniae infection in children with clinical diagnosis of atypical pneumonia. J Microbiol Immunol Infect.

[13] Shen XX, Qiu FZ, Shen LP, Yan TF, Zhao MC, Qi JJ, Chen C, Zhao L, Wang L, Feng ZS, Ma XJ (2019). A rapid and sensitive recombinase aided amplification assay to detect hepatitis B virus without DNA extraction. BMC Infect Dis.

[14] Yan TF, Li XN, Wang L, Chen C, Duan SX, Qi JJ, Li LX, Ma XJ (2018). Development of a reverse transcription recombinase-aided amplification assay for the detection of coxsackievirus A10 and coxsackievirus A6 RNA. Arch Virol.

[15] Chen C, Li XN, Li GX, Zhao L, Duan SX, Yan TF, Feng ZS, Ma XJ (2018). Use of a rapid reverse-transcription recombinase aided amplification assay for respiratory syncytial virus detection. Diagn Microbiol Infect Dis.

[16] Wang RH, Zhang H, Zhang Y, Li XN, Shen XX, Qi JJ, Fan GH, Xiang XY, Zhan ZF, Chen ZW, Ma XJ (2019). Development and evaluation of recombinase-aided amplification assays incorporating competitive internal controls for detection of human adenovirus serotypes 3 and 7. Virol J.

[17] Aizawa Y, Oishi T, Tsukano S, Taguchi T, Saitoh A (2014). Clinical utility of loop-mediated isothermal amplification for rapid diagnosis of mycoplasma pneumoniae in children. J Med Microbiol.

[18] Ding X, Xu Z, Yin K, Sfeir M (2019). Liu cc.Dual-priming isothermal amplification (DAMP) for highly sensitive and specific molecular detection with ultralow nonspecific signals. Anal Chem.

[19] Medjo B, Atanaskovic-Markovic M, Radic S, Nikolic D, Lukac M, Djukic S (2014). Mycoplasma pneumoniae as a causative agent of communityacquired pneumonia in children: clinical features and laboratory diagnosis. Ital J Pediatr.

[20] Xiao L, Ptacek T, Osborne JD, Crabb DM, Simmons WL, Lefkowitz EJ, Waites KB, Atkinson TP, Dybvig K (2015). Comparative genome analysis of mycoplasma pneumoniae. BMC Genomics.

[21] Dumke R, Schurwanz N, Lenz M, Schuppler M, Luck C, Jacobs E (2007). Sensitive detection of mycoplasma pneumoniae in human respiratory tract samples by optimized real-time PCR approach. J Clin Microbiol.

[22] Ursi D, Dirven K, Loens K (2003). Detection of *Mycoplasma pneumoniae* in respiratory samples by real time PCR using an inhibition control. J Microbiol Methods.

[23] Winchell JM, Thurman KA, Mitchel SL (2008). Evaluation of three real-time PCR assays for detection of mycoplasma pneumoniae in an outbreak investigation. J Clin Microbiol.

[24] Zhou Z, Li X, Chen X, Yao L, Pan C, Huang H, Luo F, Zheng X, Sun X, Tan F (2015). Comparison of P1 and 16S rRNA genes for detection of mycoplasma pneumoniae by nested PCR in adults in Zhejiang, China. J Infect Dev Ctries.

[25] Kakuya F, Kinebuchi T, Fujiyasu H, Tanaka R, Kano H (2014). Genetic point-of-care diagnosis of mycoplasma pneumoniae infection using LAMP assay. Pediatr Int.

[26] Yuan X, Bai C, Cui Q, Zhang H, Yuan J, Niu K, Feng Y, Jin X, Li P, Liu H (2018). Rapid detection of mycoplasma pneumoniae by loop-mediated isothermal amplification assay. Medicine (Baltimore).

[27] Saito R, Misawa Y, Moriya K, Koike K, Ubukata K, Okamura N (2005). Development and evaluation of a loop-mediated isothermal amplification assay for rapid detection of mycoplasma pneumoniae. J Med Microbiol.

